# Cross-Sectional Study of Hepatitis A Virus Infection in the Pantanal Population before Vaccine Implementation in Brazil: Usage of Non-Invasive Specimen Collection

**DOI:** 10.3390/ijerph120707357

**Published:** 2015-06-30

**Authors:** Renata Santos Tourinho, Adilson José de Almeida, Livia Melo Villar, Paula Guerra Murat, Gina Jonasson Mousquer Capelin, Ana Rita Coimbra Motta Castro, Vanessa Salete de Paula

**Affiliations:** 1 Laboratório de Desenvolvimento Tecnológico em Virologia, Instituto Oswaldo Cruz-FIOCRUZ, Cx Postal 926. Av., Brasil 4365, CEP: 21045-900 Rio de Janeiro/RJ, Brazil; E-Mail: vdepaula@ioc.fiocruz.br; 2 Laboratório de Hepatites Virais, Instituto Oswaldo Cruz-FIOCRUZ, Cx Postal 926. Av., Brasil 4365, CEP: 21045-900 Rio de Janeiro/RJ, Brazil; E-Mails: adilsonjoal@ioc.fiocruz.br (A.J.A.); lvillar@ioc.fiocruz.br (L.M.V.); 3 Departamento de Farmácia Bioquímica, Universidade Federal de Mato Grosso do Sul, Campo Grande, MS, Cx Postal 549, Brazil; E-Mails: paulamurat@hotmail.com (P.G.M.); gina.jm@hotmail.com (G.J.M.C.); arcm.castro@hotmail.com (A.R.C.M.C.)

**Keywords:** hepatitis A, epidemiology, oral fluid, vaccine

## Abstract

Population-based prevalence studies are essential tools for screening of hepatitis A and provide important data on susceptible groups. However, surveillance in isolated communities is difficult because of the limited access to these areas and the need for blood sample collection. This study aimed to determine the anti-HAV prevalence using oral fluid samples to provide an alternative tool for epidemiological studies that might be useful for vaccination-related decisions. The study population was composed of 224 volunteers from South Pantanal, aged 3 to 86 years old. This study was performed using oral fluids, previously standardized for anti-HAV antibody detection, which were collected using a ChemBio device. Eluates were tested using modified commercial EIA to detect anti-HAV antibodies. The overall prevalence was 79.1%, corresponding to 178 reactive EIA tests out of 224 samples. The age stratified data revealed a prevalence of 47.8% between 0–10 years, 84% in 11–20 years and 91.9% in subjects older than 21 years. Results indicate that hepatitis A prevalence was higher in adolescents and adults, corroborating the literature reports. Thus, oral fluid samples could replace serum in HAV epidemiological studies in isolated communities as they are efficient at detecting anti-HAV antibodies.

## 1. Introduction

Hepatitis A is one of the most frequently reported vaccine-preventable diseases and remains endemic in many areas of the world, especially in developing countries [1]. Hepatitis A virus (HAV) has been observed in heterogeneous pockets where susceptible or exposed individuals may co-exist. Therefore, there is a threat of a small and localized outbreak of HAV infection or even a larger outbreak in such areas [2].

The association of HAV infection with poor access to sanitation and hygiene behavior patterns, age-dependent clinical outcome of the disease, and lifelong immunity determine the different patterns of HAV infection observed around the world [3,4]. In areas marked by an increased prevalence rate of hepatitis A, the infection is primarily observed among children, and most of them present with asymptomatic to self-limiting disease, generating protective immunity against hepatitis A [5]. Conversely, in areas marked by an intermediate prevalence rate for hepatitis A, a limited proportion of the adult population is reported to be immune. As a result, community-wide epidemics, resulting from person-to-person transmission may occur [5]. Finally, in areas marked by a low prevalence rate for hepatitis A, residents may be at risk for infection by travel to areas where hepatitis A is endemic and ingestion of local contaminated foods [5]. Despite the mild course of the infection during childhood, hepatitis A may cause significant morbidity and mortality among adolescents and adults [6]. Fulminant hepatitis may also develop among individuals with a predisposing underlying liver disease [7].

As a result of the epidemiological pattern transition of hepatitis A in Brazil, two distinct epidemiological patterns may be observed: the North, Northeast, and Midwest regions with intermediate endemicity of hepatitis A and the South and Southeast regions with low endemicity [8,9].This situation was highlighted in the discussion about universal mass hepatitis A vaccination program in the country. In early 2013, a vaccine against hepatitis A was incorporated into the routine children vaccination program in Brazil as a result of a cost-effective analysis to control HAV infection [5]. To determine whether the hepatitis A vaccine is effective as part of the national vaccination program, data regarding the prevalence and epidemiology of hepatitis A are essential.

In the epidemiological context, the use of oral fluids to determine HAV protection has been demonstrated to be appropriate because of its advantages and high accuracy for surveillance studies in different groups [10,11]. The advantages of oral specimen collection and evaluation of performance of several oral fluid collection devices in modified EIAs have led to increased interest in the use of oral fluid samples as a surrogate for serum samples. These aspects are very important, especially if we consider difficult-to-access areas where blood collection may be challenging, such as the Pantanal region. Pantanal is a vast wetland area, located mostly in Brazilian territory, which is of great interest for epidemiological studies [12] as its hydrography can be a source element for hepatitis A virus transmission. Nevertheless, there are no data concerning local hepatitis A virus circulation in this region. Thus, the purpose of the present study was to determine the anti-HAV prevalence using oral fluid samples to provide an alternative tool for epidemiological studies that might be useful for vaccination-related decisions.

## 2. Materials and Methods

### 2.1. Ethical Aspects

Ethical permission for collecting and testing samples was provided by the FIOCRUZ Ethical Committee (number 536/2009), and written informed consent was obtained from each participant before enrollment in the study. The specimens and questionnaires were anonymous, and feedback was provided to all participants of the study, including their hepatitis results. All unprotected participants were advised to be vaccinated against hepatitis A.

### 2.2. Sample Collection and Processing

Matched serum and oral fluid samples were collected from each participant. Five milliliters (mL) of peripheral blood was drawn by venipuncture using hypodermic needles and multiple sterile vacuum blood collection tubes (Vacutainer system, Becton, Dickinson and Company, Franklin Lakes, NJ, USA). Subsequently, the samples were centrifuged at 1300 g at 25 °C for 5 min, and the sera were stored at −20 °C. Oral fluid samples were obtained by means of a commercial device, ChemBio^®^ (ChemBio Diagnostic Systems Inc., New York, NY, USA), which consists of a sponge swab attached to a handle with a plastic tube containing 500 μL of a preservative solution. The swab was rubbed along the teeth/gum line for a minute. The collected oral fluid was concentrated at the bottom of a plastic tube after centrifugation at 1300 g at 25 °C for 10 min and stored at 2–8 °C until analysis.

### 2.3. Sample Screening

Total anti-HAV antibodies were detected by using a commercially available, solid-phase enzyme immunoassay (EIA) based on the principle of immunocapture (ImmunoComb^®^II HAV Ab, Orgenics, Israel). The solid phase is a comb composed of 12 projections, each one being sensitized at two positions: an upper spot with a monoclonal anti-HAV antibody (internal control) and a lower spot with rabbit anti-human IgG and IgM antibodies.

The test was performed according to the manufacturer's instructions and adapted for oral fluid samples by adding 25 µL of oral fluid without sample diluents, as reported by Tourinho *et al.* [1]. The test results were visible as gray-blue spots on the surface of the projections, and the visual results were determined semi-quantitatively by comparing the intensity of the color of the lower spot on each projection with the color scale provided by the manufacturer. The samples results were classified according to the cut-off point (10 IU/L) of the test. A spot with an intensity greater to or equal than the cut-off point indicated the presence of protecting anti-HAV levels. A spot with an intensity slightly less than that of the cut-off was considered an equivocal result, and the sample was retested. A spot with a lower intensity than that of the cut-off was considered negative. The ImmunoComb^®^II HAV Ab assay has a limit of detection of 10 IU anti-HAV antibodies/L, which is regarded as the minimum concentration of anti-HAV antibodies that indicates immunization has occurred. All of the samples were assayed three times, and identical visual readings for HAV were consistently observed by multiple investigators (three).

### 2.4. Studied Population

**Figure 1:**
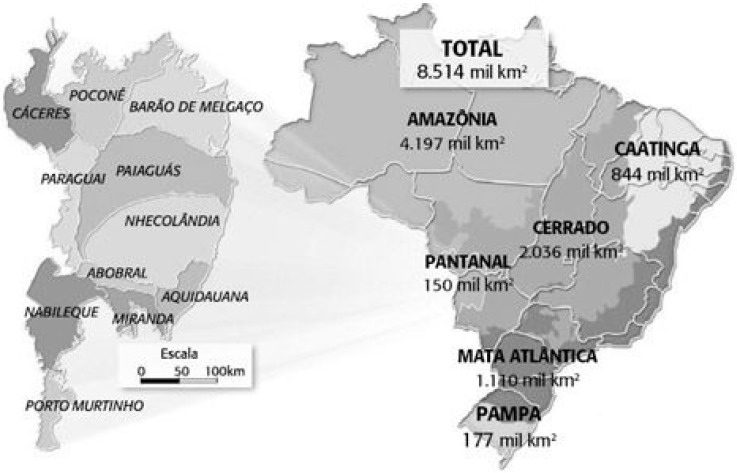
Sub-regions of the Brazilian Pantanal area (adapted from Globo Rural-Edition^©^ 288-October 2009).

Encompassing approximately 140,000 km^2^ across the territories of Brazil, Bolivia, and Paraguay, the Pantanal region is one of the world’s largest freshwater wetland ecosystems [12]. In Brazil, where 85% of the total area of Pantanal is located, 65.5% of the area is situated in the State of Mato Grosso do Sul (MS), and it is known as South Pantanal [13,14]. The region is ecologically classified into sub-regions that differ in the degree of veg­etation, flooding and physiognomy [15,16,17] (Figure 1).

This study was performed in four isolated communities of the Paraguay river basin sub-region, in areas that are 661 km far from the city of Campo Grande (MS). This region is sparsely populated, and it is characterized by wetlands that hinder access to the coastal communities; access is only available by boat. The whole population of the study area is estimated to be 691 individuals. The survey was conducted between April 2010 and June 2010, and a total of 224 paired serum and oral fluid samples were collected using a non-probability sampling method from all consenting occupants of households. The samples were placed into a cool box and returned to the laboratory after 15 days of collection for total anti-HAV screening test. The sociodemographic characteristics of each member of the study were obtained by means of questionnaires. No individual had a history of hepatitis A vaccination.

### 2.5. Statistical Analysis

Data are presented as frequencies. The performance of the laboratory tests with the collected oral fluid samples was determined by comparing the sensitivity, specificity, and positive and negative predictive values and their respective 95% confidence intervals (95% CI) with the serum results, which were used as a gold standard control. The linear and weighted kappa (***k***) statistic was used to evaluate the rate of agreement between the oral fluid and serum anti-HAV antibody status. According to the strength of the agreement, the ***k*** value was interpreted as follows [18]: <20%, poor; 21% to 40%, fair; 41% to 60%, moderate; 61% to 80%, good; and 81% to 100%, very good. To compare proportions, the chi-square (χ²) test for independence with Yate’s continuity correction, χ² for trends, and Fisher’s exact test (when appropriate) were used. A two-tailed *p* < 0.05 was considered statistically significant. All analyses were performed with MedCalc for Windows, version 8.1.0.0 (MedCalc Software, Mariakerke, Belgium) and GraphPad InStat version 3.05 software (GraphPad Software, La Jolla, CA, USA).

## 3. Results

### 3.1. Study Design and Patients

This study was a population-based cross-sectional survey, and it was conducted in four isolated communities living in difficult-to-access areas of the Paraguay River basin, South Pantanal, Brazil: Serra do Amolar/SãoLourenço, Paraguay Mirim, Porto da Manga, and Passo do Lontra.

### 3.2. Sociodemographic Characteristics of the Studied Population

In this seroepidemiological survey, a total of 224 matched serum and oral fluid samples were obtained from volunteers, 100 (43.9%) of which were female and 124 (56.1%) male. The age of the study population ranged from 3 to 86 years, with a mean age of 26.91 ± 17.35 years. Because of the lack of data on anti-HAV prevalence in the studied communities, as many volunteers as possible were recruited from among the local residents (Table 1).

**Table 1:** Sociodemographic characteristics of the study subjects (*n* = 224).

*Variable*	*Studied Population (n)*	*Studied Population (%)*
***Age group (years)***		
0–10	46	20.53%
11–20	50	22.32%
21–30	47	20.98%
31–40	26	11.61%
41–50	29	12.95%
>50	26	11.61%
***Gender***		
Female	100	43.90%
Male	124	56.10%
***Race/Ethnicity***		
Caucasian	67	29.91%
Brown	55	29.00%
Black	41	18.30%
Amerindian	37	16.52%
Asian	8	3.57%
Not reported	6	2.70%
***Educational level***		
None	19	8.48%
First grade	156	69.60%
Second grade	32	14.29%
Graduated	14	6.25%
Not reported	3	1.38%
***Family income***		
1 minimun salary	107	47.80%
2 minimun salary	95	42.40%
3 minimun salary	19	8.50%
Not reported	3	1.30%
***Community***		
Serra do Amolar/São Lourenço	45	20.09%
Paraguai-Mirim	60	26.78%
Porto da Manga	23	10.27%
Passo do Lontra	96	42.86%

### 3.3. Anti-HAV Antibodies Detection in Serum and Oral Fluid Samples

Total anti-HAV antibodies were detected in 181/224 serum samples by using a commercial immunoassay, ImmunoComb II HAV Ab (Orgenics), corresponding to a hepatitis A seroprevalence of 80.8%. The prevalence of total anti-HAV antibodies in oral fluid was 79.01%, corresponding to 177 reactive samples. Analysis of the test performance revealed the following results: sensitivity of 97.24% (95% CI: 0.936 to 0.991), specificity of 97.67% (95% CI: 0.877 to 0.999), positive predictive value of 99.44% (95% CI: 0.968 to 0.999), negative predictive value of 89.36% (95% CI: 0.768 to 0.964), and Kappa coefficient of 91.7% (95% CI: 0.851 to 0.982).

### 3.4. Factors Associated with Total Anti-HAV Positivity

According to bivariate analysis, age and ethnicity were found to be statistically associated with total anti-HAV positivity in serum samples. However, only age was a factor statistically related to anti-HAV positivity in oral fluid samples (Table 2).

**Table 2:** Factors associated with anti-HAV antibodies positivity in serum and oral fluid (Chembio^®^) samples from individuals living in South Pantanal, Brazil (*n* = 224).

*Variable*	*Serum*			*Oral Fluid (ChemBio^®^)*		
	*Positive (n = 181)*	*Negative (n = 43)*	*p*	*Positive (n = 177)*	*Negative (n = 47)*	*p*
***Age (years), median (interval)***	27 (5.0–86.0)	10 (3.0–72.0)	**<0.0001**	26 (5.0–86.0)	11 (3.0–77.0)	**<0.0001**
***Gender, n (%)***			0.9175			0.9953
Male	101 (55.8)	23 (53.4)		98 (53.3)	26 (55.3)	
Female	80 (44.2)	20 (46.6)		79 (46.7)	21 (44.7)	
***Race/Ethnicity, n (%)***			**0.0312**			0.2797
Caucasian	33 (18.2)	14 (32.6)		51 (28.8)	16 (34.0)	
Brown	58 (32.0)	7 (16.3)		57 (32.2)	8 (17.0)	
Black	53 (29.3)	8 (18.6)		32 (18.1)	9 (19.3)	
Amerindian	26 (14.4)	11 (25.6)		26 (14.7)	11 (23.4)	
Asian	6 (3.3)	2 (4.6)		6 (6.4)	2 (4.2)	
NR ^a^	5 (2.8)	1 (2.3)		5 (2.8)	1 (2.1)	
***Domestic residents, n (%)***			0.1589			0.7919
0–1	17 (9.4)	5 (11.6)		16 (9.0)	6 (12.8)	
2–3	56 (30.9)	4 (9.3)		53 (29.9)	7 (14.9)	
4–5	41 (22.7)	14 (32.6)		40 (22.6)	15 (31.9)	
6–7	33 (18.2)	9 (20.9)		32 (18.1)	10 (21.3)	
>7	33 (18.2)	10 (23.3)		35 (19.8)	8 (17.0)	
NR	1 (0.6)	1 (2.3)		1 (0.6)	1 (2.1)	
***Educational level, n (%)***			0.8396			0.7248
None	14 (7.7)	5 (11.6)		14 (7.9)	5 (10.7)	
First grade	127 (70.2)	29 (67.5)		126 (71.2)	30 (63.8)	
Second grade	28 (15.5)	4 (9.3)		25 (14.1)	7 (14.9)	
Graduated	10 (5.5)	4 (9.3)		10 (5.6)	4 (8.5)	
NR	2 (1.1)	1 (2.3)		2 (1.2)	1 (2.1)	
***Familiar income (minimum salary), n (%)***			0.7331			0.8599
≤1	82 (45.3)	25 (58.1)		81 (45.7)	26 (55.3)	
2	85 (47.0)	10 (23.3)		82 (46.3)	13 (27.7)	
3	12 (6.6)	7 (16.3)		12 (6.8)	7 (14.9)	
NR	2 (1.1)	1 (2.3)		2 (1.2)	1 (2.1)	
***Drinking water (source), n (%)***			0.2302			0.4174
Untreated (river)	56 (30.9)	11 (25.6)		56 (31.6)	11 (23.4)	
Treated (river) ^b^	107 (59.2)	30 (69.8)		104 (58.6)	33 (70.2)	
Bottled (mineral water)	16 (8.8)	1 (2.3)		5 (2.8)	2 (4.2)	
NR	2 (1.1)	1 (2.3)		2 (1.2)	2 (4.2)	
***History of hepatitis A, n (%)***			0.0903			0.2506
No	161 (89.0)	42 (97.7)		159 (89.8)	44 (93.7)	
Yes	16 (8.8)	0 (0.0)		15 (8.5)	1 (2.1)	
NR	4 (2.2)	1 (2.3)		3 (1.7)	2 (4.2)	
***Community, n (%)***			0.0562			0.1157
Passo do Lontra	81 (44.8)	15 (34.2)		77 (43.5)	19 (40.4)	
Porto da Manga	22 (12.2)	1 (2.3)		22 (12.4)	1 (2.1)	
Paraguai-Mirim	43 (23.7)	17 (39.5)		43 (24.3)	17 (36.2)	
Serra do Amolar/São Lourenço	35 (19.3)	10 (23.3)		35 (19.7)	10 (21.3)	

**^a^** Not Reported; **^b^** including treated water by chlorination, filtration and boiling.

The seroprevalence of HAV infection ranged from 70.2% in Caucasian individuals to 89.2% in brown subjects. The positivity for these antibodies in serum was higher in brown and black and subjects, 89.2% and 86.9%, respectively, compared with the other ethnicities of the study.

**Figure 2:**
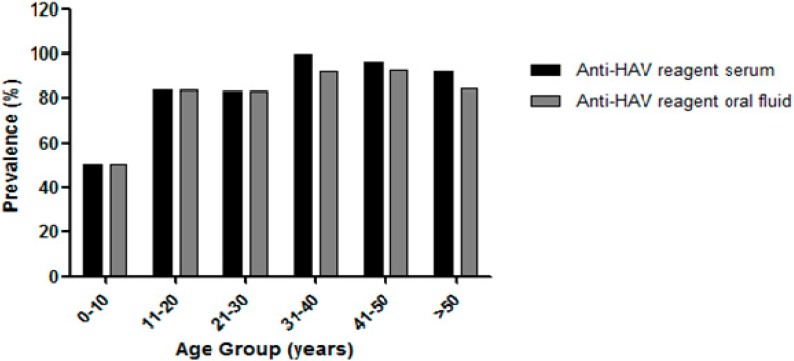
Total anti-HAV prevalence in serum and oral fluid by age group from individuals linving in difficul-to-access areas of South Pantanal, Brazil.

The proportions of anti-HAV positivity were considered to be similar in both types of biological samples by age group, with a maximum difference of 7.7% in the age group of 31–40 years old. The seroprevalence progressively increased from 50% in children 0–10 years up to 100% among individuals aged 31–40 years. A similar trend was observed for oral fluid samples, where disease prevalence ranged from 50% in children 0–10 years to 93.10% among individuals who were 41–50 years old. There was a significant increase in prevalence, approximately 40%, between the ages of 0–10 and 11–20 years of age, for both types of clinical specimens (Figure 2).

**Figure 3:**
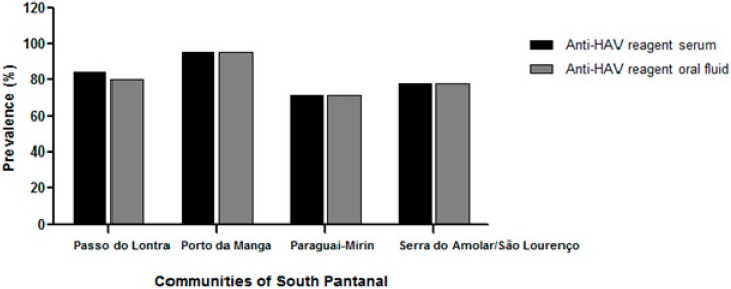
Total anti-HAV prevalence in serum and oral fluid by communites from individuals linving in difficul-to-access areas of South Pantanal, Brazil.

A difference in the anti-HAV positivity rate among the communities studied, which ranged from 71.67% (43/60) in the community of Paraguay-Mirim to 95.65% (22/23) in Porto da Manga, was also observed. The prevalence of HAV infection was higher in communities closer to urban centers, such as Passo do Lontra (84.38%) and Porto da Manga (95.65%), than in most remote and difficult-to-access communities, such as Paraguay-Mirim (71.67%) and Serra do Amolar/São Lourenço (77.78%) (Figure 3). Despite this variability, anti-HAV positivity was not significantly associated with a particular community.

## 4. Discussion

In Brazil, HAV infection accounts for the majority of viral hepatitis notifications. Changes in the epidemiological patterns have been described in some regions due to improvements in sanitation and socioeconomic conditions. Nevertheless, all three patterns can be identified even within a particular geographical region [8]. HAV infection has an intermediate prevalence in Brazil, and it is considered endemic to the country [19].

The issue of implementing a national vaccination program against hepatitis A in Brazil has been widely discussed in view of the difficulty of establishing a unified project in an area of continental dimensions and macro-regions with distinct epidemiological profile of the disease. In Brazil, HAV available vaccine is imported, and it has been recently evaluated for inclusion in the childhood vaccination schedule. For determination of national policies on vaccination, the results of epidemiological studies and cost-benefit must be carefully considered and the impact on public health must be balanced [2,20].

For epidemiological monitoring purposes, the use of oral fluid samples is of great importance because, as shown by different authors [21,22,23,24,25,26] this specimen type has many clinical advantages and appears to be accurate enough to be used in such situations. However, many of these studies have proposed the use of oral fluid as an alternative tool for blood samples. If the benefits of such clinical specimen have been demonstrated, and its efficiency in enzyme immunoassays has sometimes proven, why not use it as a substitute for blood collection?

As demonstrated in this study, oral fluid samples may, in fact, serve as a substitute for blood collection. However, it is important to select the most appropriate device for sample collection and field studies [1,27], providing accurate information to determine the epidemiological profile, the need for immunization and disease control strategies [28].

To determine the efficiency oral fluid samples and its applicability in field research as a substitute for serum, the first investigation of HAV infection in difficult-to-access communities was held in the South Pantanal. Using samples collected from different individuals belonging to these communities, we observed a close relationship between the HAV prevalence in oral fluid samples collected with ChemBio^®^ (79.01%) device and HAV seroprevalence (81.25%). The agreement between the results of oral fluid and the “gold standard” (serum) was 97.32% (sensitivity = 97.24% and specificity = 97.67%).

A high overall anti-HAV prevalence rate (81.25%) was found compared with that observed in the general population (55.7%) in the South and Southeast regions of Brazil. However, this rate was lower than the rate found in the North (92.8%) and more similar to the rate obtained in the Northeastern region of Brazil (76.5%) [29].

Although some studies have demonstrated that urban populations have lower rates of HAV infection than rural populations [30,31,32], in this study, communities closer to urban centers had a higher rate of HAV exposure than the rate observed among individuals from the rural areas (91.65% and 84.38% *versus* 71.67% and 77.78%, respectively). Similarly, Almeida and colleagues [33] found a higher HAV prevalence in urban areas (87.4%) compared with rural settlement areas of Cavunge (79.7%), a semiarid region of Bahia state in Northeast Brazil.

Furthermore, in the present investigation, people living in these communities reported having a low income. Despite the fact that anti-HAV positivity was not significantly associated with water source, the inhabitants of these rural areas do not have adequate facilities for sanitation and use river water for washing and consumption as well as for their personal hygiene. Urban communities are located near the hotel industry and farms, where poor sanitation conditions persist, and environmental sanitation projects either do not exist or are still under construction. Apart from sanitation facilities and hygiene conditions, the residents’ houses are located close to each other, and the high population density in low-income urban communities can contribute to the spread of the hepatitis A virus.

The anti-HAV seroprevalence was significantly associated with age. Stratification by age revealed that although the overall prevalence was 81.25%, only 50% of children aged 0–10 years are immune to the disease. This rate is higher than that observed by De Alencar Ximenes and colleagues [8] in the capitals of the Northeast and Midwest regions of Brazil, 32% and 34%, respectively. However, the prevalence is lower (60%) than in individuals younger than 10 years in the Amazon region [34]. Data from these studies demonstrate a low prevalence in this age group in these regions.

Despite the prevalence of HAV infection having been linked to race/ethnicity, this association appears more to reflect a regional characteristic.

The combination of the collection and preservation of samples with the stabilizers in ChemBio^®^ device methodology is considered as an important strategy to avoid problems of rapid degradation during antibody storage, as reported by Gröschl and colleagues [35] for other collectors.

## 5. Conclusions

The use of oral fluid specimens is a helpful tool for understanding the hepatitis A prevalence in areas where no data are available and might be useful for vaccination-related decisions since it is efficient in detecting susceptible individuals.
